# Long-term effects of linear *versus* macrocyclic GBCAs on gene expression in the central nervous system of mice

**DOI:** 10.1186/s41747-024-00546-x

**Published:** 2025-01-10

**Authors:** Chuanbing Wang, Yuxia Tang, Jiajia Tang, Jie Zhang, Siqi Wang, Feiyun Wu, Shouju Wang

**Affiliations:** Laboratory of Molecular Imaging, Department of Radiology, The First Affiliated Hospital of Nanjing Medical University, Nanjing, Jiangsu China

**Keywords:** Animals, Central nervous system, Contrast media, Gadolinium, Gene expression

## Abstract

**Background:**

We examined chronic gadolinium retention impact on gene expression in the mouse central nervous system (CNS) after injection of linear or macrocyclic gadolinium-based contrast agents (GBCAs).

**Methods:**

From 05/2022 to 07/2023, 36 female mice underwent weekly intraperitoneal injections of gadodiamide (2.5 mmol/kg, linear), gadobutrol (2.5 mmol/kg, macrocyclic), or saline. Mice were sacrificed on day 29 or 391 after a 1-year washout. Assessments included magnetic resonance imaging (MRI), mechanical hyperalgesia tests, and inductively coupled plasma mass spectrometry to measure gadolinium levels. Ribonucleic acid (RNA) sequencing and bioinformatic analyses identified differentially expressed genes (DEGs), with validation by quantitative reverse transcription polymerase chain reaction (qRT-PCR) and western blot (WB).

**Results:**

Post-gadodiamide, MRI showed increased signal intensity in the deep cerebellar nuclei (pre, 0.997 ± 0.006 *versus* post, 1.086 ± 0.013, *p* < 0.001). Mechanical hyperalgesia tests indicated transient sensory changes. After 1-year, gadolinium retention was noted in the brain (5.92 ± 0.32 nmol/kg) and spinal cord (1.23 ± 0.66 nmol/kg) with gadodiamide, compared to saline controls (0.06 ± 0.02 nmol/kg in brains and 0.28 ± 0.06 nmol/kg in spinal cords). RNA sequencing identified 17 shared DEGs between brain and spinal cord in the gadodiamide group on day 391, with altered *Hmgb2* and *Sgk1* expression confirmed by qRT-PCR and WB. Reactome pathway analysis showed enrichment in neuroinflammation pathways. No DEGs were detected in brains on day 29.

**Conclusion:**

Chronic gadolinium deposition from repeated linear GBCA but not macrocyclic administration causes significant gene expression alterations in the mouse CNS, particularly affecting neuroinflammation pathways.

**Relevance statement:**

This study examined the long-term impact of chronic gadolinium retention on gene expression in the mouse CNS, uncovering significant changes associated with neuroinflammation pathways after repeated administration of linear GBCA, but not with macrocyclic GBCA. These findings highlight the importance of further research on the long-term safety of linear GBCA in medical imaging.

**Key Points:**

- Chronic gadolinium retention alters gene expression in the mouse central nervous system.
- Significant neuroinflammatory pathway changes were observed after linear gadodiamide exposure.
- MRI showed increased signal intensity in deep cerebellar nuclei after gadodiamide injection.

**Graphical Abstract:**

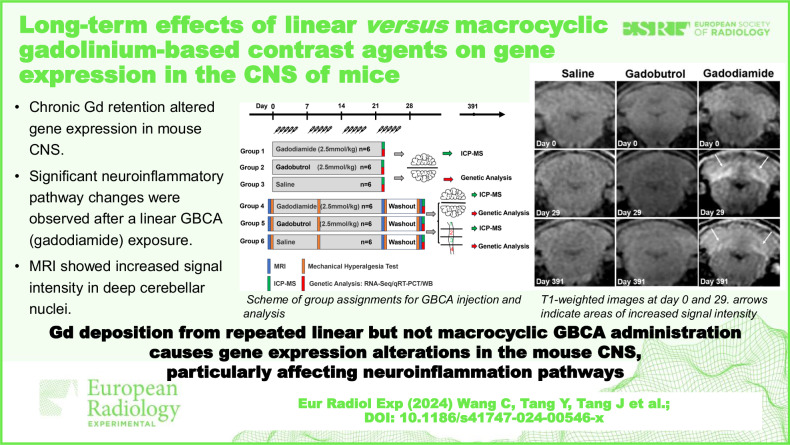

## Background

Since the approval of the first gadolinium-based contrast agent (GBCA) by the Food and Drug Administration in 1988, GBCAs have revolutionized medical imaging, becoming an indispensable tool in clinical MRI examinations [1]. Their widespread adoption, with over 50 million doses administered worldwide annually, was largely attributed to their perceived safety among patients with normal renal function [2].

However, this perception is being challenged. Emerging research indicates that GBCA injections can lead to gadolinium deposition in the brain, even in subjects with normal renal function and intact brain-blood barrier [3]. Alarmingly, animal studies have highlighted that linear GBCAs can linger in brain tissues for durations exceeding a year [4, 5]. Moreover, certain gadolinium ions in linear GBCAs are found to dissociate from their chelated states, binding with biomacromolecules [1, 5].

The implications of these findings are profound. While no overt toxicity from chronic residual GBCAs has been documented in clinical or animal models thus far, the potential for subtle tissue alterations remains a significant concern. The discovery of gadolinium deposits in the nuclei of neurons in patients merits additional investigation into the genotoxic potential of dechelated gadolinium [6]. There’s a growing realization that subtle molecular, genetic, and protein-level changes might be occurring beneath the radar of conventional examination techniques. For instance, while El Hamrani et al [7] reported no discernible metabolic biomarker changes in brain tissues with chronic residual linear GBCAs using *in vivo* magnetic resonance spectroscopy, Romano et al [8] unveiled alterations in metabolic pathways in mouse brains, cerebellum, and liver tissues through metabolomics techniques.

In light of these revelations, our study seeks to bridge this knowledge gap. We aim to ascertain whether chronic residual GBCAs can induce genetic level changes in the central nervous system (CNS) of healthy mice. Utilizing ribonucleic acid (RNA) sequencing, we analyzed the transcriptomes of central nervous tissues in mice before and after 1-year washout period following repeated gadodiamide or gadobutrol injections. To bolster our findings, representative differentially expressed genes (DEGs) were further validated using quantitative reverse transcription polymerase chain reaction (qRT-PCR) and western blot (WB).

## Methods

This prospective animal study was conducted from May 2022 to July 2023 after approval by the institutional animal research committee. All animal experiments followed the ethical guidelines of our institution.

### Animals

Thirty-six 6-week-old female BALB/c mice weighing 15–20 g were used. The mice were housed at 20–22 °C with a 12-h light-dark cycle.

### GBCA injection

The mice were randomly divided into six groups (*n* = 6 per group):

- group 1 received intraperitoneal injections of the linear GBCA gadodiamide (Omniscan, GE Healthcare, Oslo, Norway) at 2.5 mmol/kg five times per week for four consecutive weeks and were euthanized on day 29;
- group 2 received intraperitoneal injections of the macrocyclic gadobutrol (Gadovist, Bayer AG, Berlin, Germany) at 2.5 mmol/kg five times per week for four consecutive weeks and were euthanized on day 29;
- group 3 received saline injections on the same schedule a group 1 and group 2 and were euthanized on day 29;
- group 4 received the same gadodiamide injection regimen as group 1 and were euthanized after a 1-year washout period on day 391;
- group 5 received the same gadobutrol injection regimen as group 2 and were euthanized after a 1-year washout period on day 391;
- group 6 received saline injections on the same schedule as group 4 and group 5 and were euthanized on day 391.

**Fig. 1:**
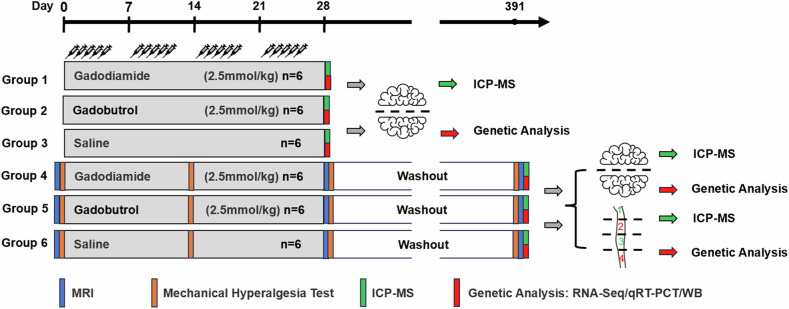
Global scheme of group assignments for gadolinium-based contrast agent injections and the following analysis

All injections were performed under isoflurane anesthesia (1–3% inhalation). Mice and organs were assigned to different analyses according to Fig. 1.

### Magnetic resonance imaging (MRI) study

Mice in groups 4, 5, and 6 underwent MRI at baseline (day 0, before injections), day 29 and day 391, using a 3-T clinical MRI system (GE Healthcare 750 W) with a specialized animal coil (Shanghai Chenguang Medicine, China). Imaging was performed under isoflurane anesthesia (1.5%), with animals taped prone to minimize motion. A T1-weighted three-dimensional gradient-echo sequence was conducted with the following parameters: repetition time 40 ms, echo time 2.15 ms, flip angle 45°, field of view 80 × 64 mm, matrix 384 × 320, 6 excitations, section thickness 1 mm. The total scan time was 19:35 min. Animals were placed on a heated water pad to maintain body temperature.

### Image analysis

Signal intensity (SI) changes were measured as described previously. Two regions of interest were manually drawn to cover the entire bilateral deep cerebellar nuclei (DCN) on the most representative section. The mean SI of the two regions of interest was defined as SI_DCN_. The SI of the region of interest covering the full cerebellar hemisphere, excluding the DCN, was defined as SI_CH_. The DCN SI ratio was calculated as SI_DCN_/SI_CH_. Two blinded, experienced radiologists independently performed all image analyses.

### Mechanical hyperalgesia test

Mice in groups 4, 5, and 6 underwent testing for mechanical hyperalgesia at baseline and on days 14, 29, and 391. After a 30-min adaptation period in a chamber with a wire grid floor, the frequency of paw withdrawal was evaluated by applying a 0.6 g von Frey filament to the plantar surface of the hind paw. Testing consisted of poking the hind paw five times, with a 5-min interval between pokes. The paw withdrawal frequency was defined as the number of withdrawals per trial. The mean of the five trials was calculated.

### Inductively coupled plasma mass spectrometry (ICP-MS)

The brains of mice in group 1, 2, and 3 as well as the brains and spinal cords of mice in group 4, 5, and 6 were collected immediately after euthanasia. The brain was bisected along the midsagittal plane. One hemisphere was used for ICP-MS analysis and the other for expression analysis. To minimize differences between the upper and lower spinal cord, the spinal cord was divided into 4 equal segments from rostral to caudal. Spinal cord segments 1 and 3 were used for ICP-MS, while segments 2 and 4 were used for expression analysis. The tissues were weighted and digested by aqua regia. The amount of Gd was determined using the Agilent ICP-MS 7700 instrument (Agilent, CA, USA).

### RNA sequencing analysis

Total RNA was extracted from the brain and spinal cord samples using Trizol reagent. RNA purity was assessed by NanoPhotometer and RNA integrity was evaluated using an Agilent Bioanalyzer. Only RNA samples with an A260/A280 ratio between 1.8 and 2.0 and RNA integrity number ≥ 8 were used. mRNA libraries were constructed using a NEBNext® UltraTM RNA Library Prep Kit and sequenced on a DNBSEQ-T7 platform to generate 150 bp paired-end reads. Reads were mapped to the mouse reference genome Mus_musculus. GRCm38.99 using HISAT2 aligner. Gene expression was quantified with HTSeq v0.6.0 using Ensembl gene annotation version 97. Differential expression analysis was performed with DESeq2 to identify significantly dysregulated protein-coding genes between conditions, using an adjusted *p*-value cutoff of 0.05, absolute log2 fold change greater than 1, and coefficient of variation within each group less than 0.5.

### Quantitative reverse transcription polymerase chain reaction (qRT-PCR)

The differential expression of *Hmgb2* and *Sgk1* was validated by qRT-PCR. Total RNA was reverse transcribed using the PrimeScript RT Master Mix. qRT-PCR was performed on an ABI Step one plus Real-time-PCR system using Real-time-PCR Master Mix (SYBR Green). The PCR cycling conditions were: 95 °C for 5 min, followed by 40 cycles of 95 °C for 15 s, 60 °C for 20 s, and 72 °C for 40 s. Target gene expression was normalized to the housekeeping gene *Gapdh*. Relative expression was calculated using the ΔΔCt method. The primer sequences for qRT-PCR are shown in Table 1. For each group, three mice were randomly selected for qRT-PCR validation.

**Table 1:** Primer sequences of target genes

Gene	Forward primer	Reverse primer
*Hmgb2*	AAAAGACCCCAATGCTCCGAA	CTCAGACCACATCTCACCCAG
*Sgk1*	AAGCACCCTTACCTACTCCAGA	ATCGTTCAGGCCCATCCTTCT
*Gapdh*	CCCAGCTTAGGTTCATCAGG	CCAAATCCGTTCACACCGAC

### Western blot analysis

Protein levels of high-mobility group box 2 (HMGB2) and serum/glucocorticoid-regulated kinase 1 (SGK1) were assessed by WB. Tissue lysates were prepared using a Protein Extraction Kit (KGP250, KeyGEN BioTECH, (Nanjing, China) according to the manufacturer’s protocol. Equal amounts of protein were separated by sodium dodecyl-sulfate polyacrylamide gel electrophoresis and transferred to polyvinylidene fluoride membranes. Membranes were blocked with 5% nonfat milk and incubated with primary antibodies for rabbit anti-HMGB2 (1:1000, 14597-1-AP), anti-SGK1 (1:1000, 28454-1-AP) and anti-glyceraldehyde-3-phosphate dehydrogenase (1:5000, KGAA002) overnight at 4 °C. After washing, membranes were incubated with horseradish peroxidase-conjugated secondary antibodies (1:5000, KGAA35) for 1 h at room temperature. Protein bands were visualized and imaged on a ChemiDoc system. Band densities were quantified using ImageJ and normalized to glyceraldehyde-3-phosphate dehydrogenase. For each group, three mice were randomly selected for qRT-PCR validation.

### Pathological analysis

The pathological analysis was conducted on paraffin sections of mouse brain tissue, following a standard protocol. Tissues were fixed, dehydrated, and embedded before sectioning. Sections were de-waxed, rehydrated, and subjected to antigen retrieval. Blocking with normal serum followed to minimize non-specific binding. Primary antibodies, rabbit anti-HMGB2 (Wuhan Sanying, catalog number 15605-1-AP) diluted at a ratio of 1:200, and rabbit anti-SGK1 (Wuhan Sanying, catalog number 28454-1-AP) diluted at a ratio of 1:400, were incubated overnight at 4 °C. After washing with phosphate-buffered saline, sections were incubated with horseradish peroxidase-conjugated secondary antibodies for an hour at room temperature. Immunoreactivity was visualized using diaminobenzidine and monitored under a microscope. Sections were counterstained with hematoxylin, mounted, and observed under a light microscope for image capture and analysis.

### Reactome pathway enrichment analysis

To understand the biological pathways altered in the differential expression data, reactome pathway enrichment analysis was performed using the online tool at the Reactome website (https://reactome.org). The list of differentially expressed protein-coding genes (DEGs) meeting the selection criteria (adjusted *p* < 0.05, absolute log2FC > 1, coefficient of variation < 0.5) was analyzed. Significantly enriched pathways were identified using an adjusted entities *p*-value cutoff of 0.05 and a minimum entity found of 5. This analysis identified key pathways and biological processes associated with the observed gene expression changes between groups.

### Statistics

The data were expressed as mean ± standard error and analyzed using R. The use of standard error was due to estimating how accurately the sample mean reflects the population mean across multiple groups. For MRI SI comparison, ICP-MS, qRT-PCR, and WB result analysis, one-way ANOVA was used. For mechanical hyperalgesia result analysis, two-way ANOVA was used. Values of *p* < 0.05 were considered as statistically significant.

## Results

### MRI

**Fig. 2:**
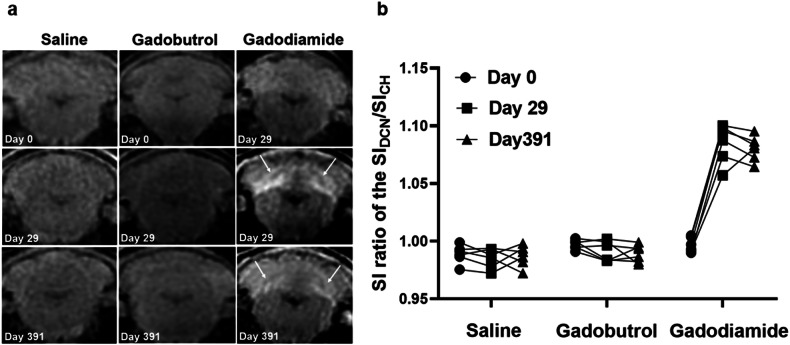
**a** Representative T1-weighted images at days 0, 29, and 391. Arrows indicate areas of increased signal intensity. **b** Quantitative signal intensity ratio of the deep cerebellar nuclei (DCN) and cerebellar hemisphere (CH) at days 0, 29, and 391

Typical non-enhanced T1-weighted images obtained from the saline, gadobutrol, and gadodiamide groups at day 0, day 29, and day 391 are shown in Fig. 2. Apparent hyperintensity was observed in the bilateral DCN of the gadodiamide group at the end of the injections (Day 29). Compared to the baseline, the SI ratio of the DCN in the gadodiamide group increased by 8.9% (1.085 ± 0.007 *versus* 0.997 ± 0.006, *p* < 0.001), while no significant difference was observed in the saline group (0.986 ± 0.008 *versus* 0.989 ± 0.008) and gadobutrol group (0.986 ± 0.008 *versus* 0.989 ± 0.008). After 1 year of washout at day 391, the SI ratio of DCN in the gadodiamide group remained high at 1.08 ± 0.004.

### Mechanical hyperalgesia

**Fig. 3:**
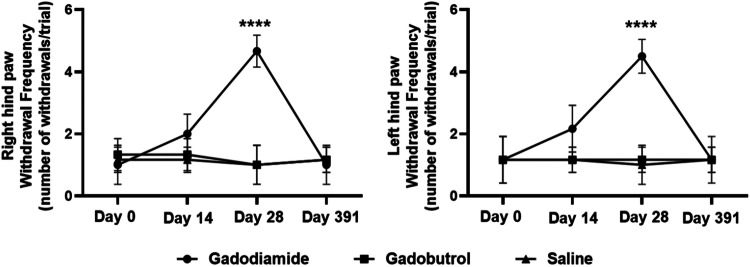
Time course of mechanical hyperalgesia (number of withdrawals per trial) in the right and left hind paw of mice treated with gadodiamide (2.5 mmol/kg), gadodiamide, gadobutrol (2.5 mmol/kg), and saline. Data are expressed as mean ± standard error of the mean (six rats per group). **** indicates *p* < 0.001

Transient mechanical hyperalgesia was observed in the gadodiamide group at day 14 (paw withdrawal frequency, left 2.2 ± 0.3, right 2.0 ± 0.3) compared to the saline group (left 1.2 ± 0.2, right 1.2 ± 0.2) and gadobutrol group (left 1.2 ± 0.2, right 1.3 ± 0.2). This effect was more remarkable at day 29 (left 4.5 ± 0.2, *p* < 0.001; right 4.7 ± 0.2, *p* < 0.001), but fully reversed after a 1-year washout (left 1.2 ± 0.2. right 1.00 ± 0.3) compared to the saline group (left 1.2 ± 0.2, right: 1.2 ± 0.2) and gadobutrol group (left 1.2 ± 0.3, right: 1.2 ± 0.2) (Fig. 3).

### Gadolinium tissue concentration

At the end of injections (day 29), the concentration of gadolinium in the brain tissues of the gadodiamide group was significantly higher than that in the saline group and gadobutrol group. After a 1-year washout (day 391), 78.8% of the gadolinium was still retained in the brain tissues of the gadodiamide group. The gadolinium concentration was significantly higher in the spinal cord tissues of the gadodiamide group after the 1-year washout compared to the saline group and gadobutrol group (Table 2).

**Table 2:** Mean gadolinium concentrations measured with inductively coupled plasma mass spectrometry

Tissues (time)	Gadodiamide	Gadobutrol	Saline	*p*-value
Brain (day 29)	7.506 ± 0.909	2.076 ± 0.134	0.056 ± 0.002	< 0.001
Brain (day 391)	5.924 ± 0.142	0.105 ± 0.011	0.060 ± 0.008	< 0.001
Spinal cord (day 391)	1.231 ± 0.294	0.336 ± 0.074	0.284 ± 0.026	< 0.010

Values represent means ± standard errors of means (gadolinium, nmol/kg)

### Differential gene expression

One brain tissue sample each from the gadodiamide and saline groups at day 29, as well as one brain tissue sample from the gadodiamide group at day 391, were excluded from DEG analysis due to poor RNA quality.

**Fig. 4:**
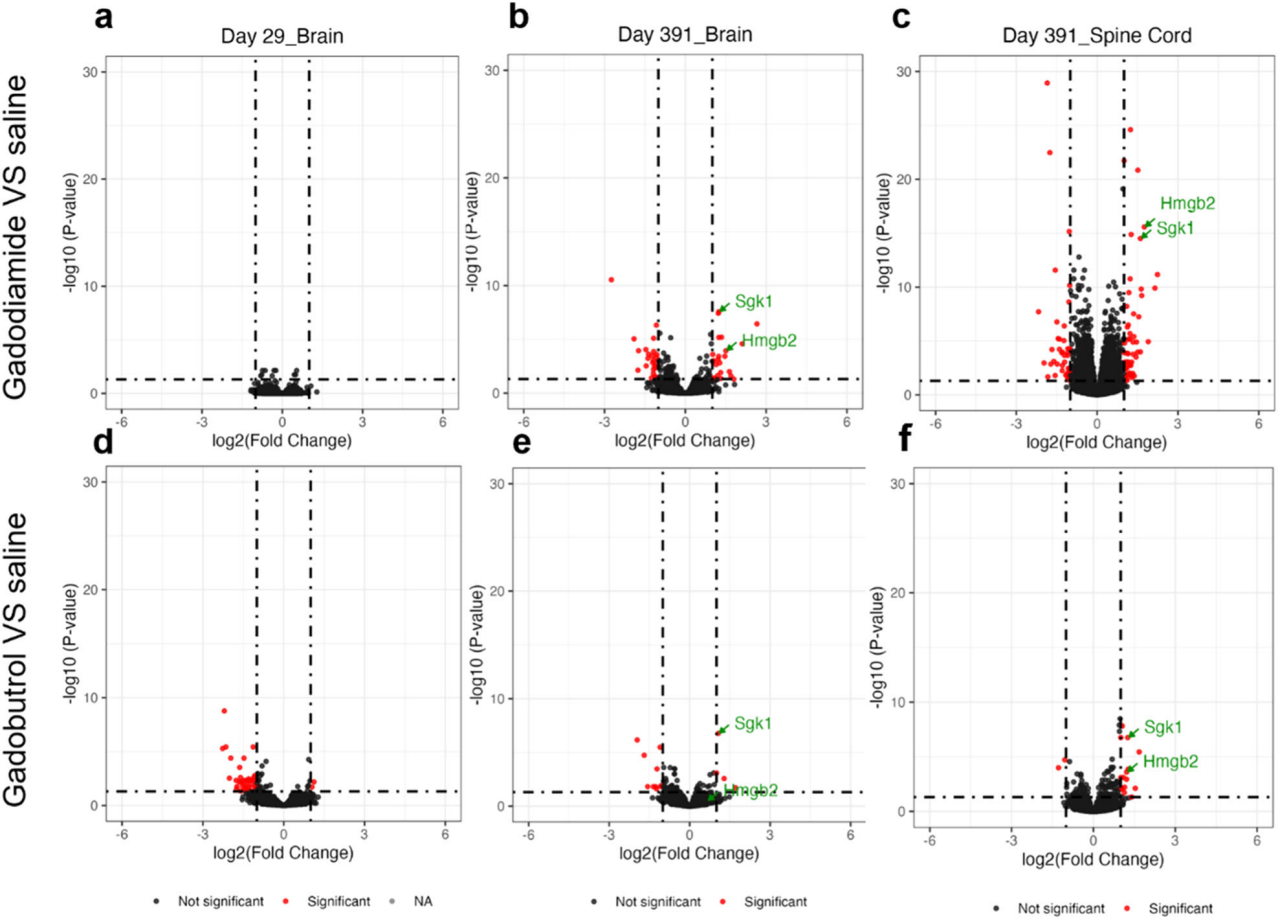
Gadodiamide *versus* saline: Volcano plots of differential gene expression for the brain on day 29 (**a**), the brain on day 391 (**b**), and the spinal cord on day 391 (**c**). Gadobutrol *versus* saline: Volcano plots for the brain on day 29 (**d**), the brain on day 391 (**e**), and the spinal cord on day 391 (**f**). Red dots indicate differentially expressed genes with adjusted *p* < 0.05 and |log2FC | > 1

In the gadodiamide-injected group, no DEGs were identified in the brain tissues at the end of the injection period, suggesting that gadodiamide does not elicit detectable alterations in the transcriptome during the acute phase of administration (Fig. 4a). However, following a 1-year washout period, significant changes in gene expression were observed. Specifically, 23 genes were found to be upregulated, and 26 genes were downregulated in the brain tissues of the gadodiamide group relative to the saline control (Fig. 4b). Similarly, in the spinal cord, 57 genes exhibited increased expression, and 40 genes showed decreased expression in the gadodiamide group compared to the saline group after the 1-year washout (Fig. 4c). A total of 17 DEGs (detailed in Supplemental Table S1) were common to both the brain and spinal cord of the gadodiamide group following the 1-year washout period. For the gadobutrol-injected group, the gene expression changes were notably less pronounced following the 1-year washout period. Specifically, in the brain tissues, only 4 genes were upregulated, and 10 genes were downregulated compared to the saline control. In the spinal cord, a modest number of changes were observed, with 2 genes showing decreased expression and 15 genes exhibiting increased expression. In contrast, at the end of the acute phase of injection, more significant alterations were detected, with 2 genes upregulated and 42 genes downregulated, potentially indicating a transient effect of gadobutrol on gene expression.

**Fig. 5:**
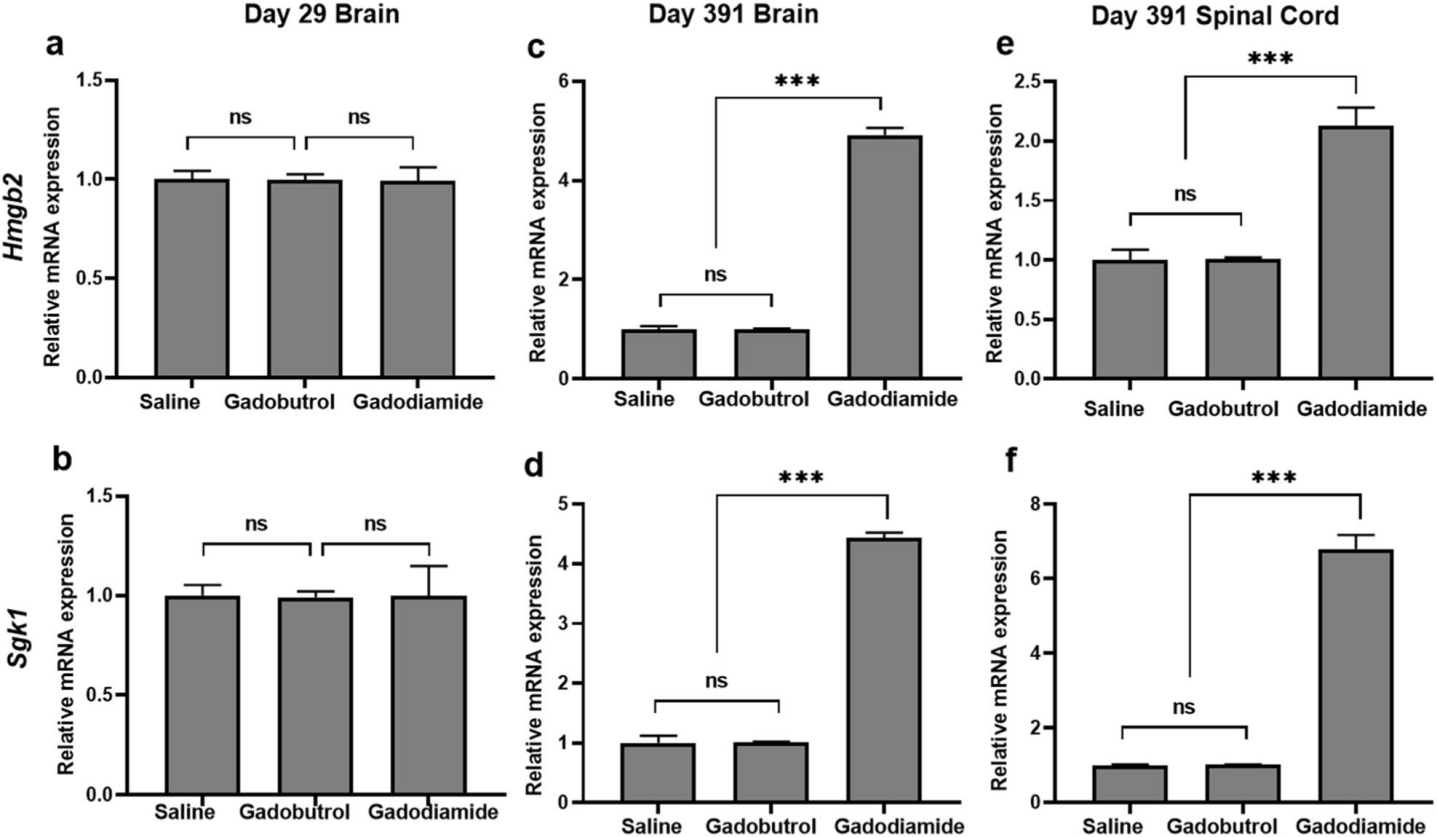
Relative mRNA expression of *Hmgb2* and *Sgk1*. Brain at day 29 (**a**, **b**), brain at day 391 (**c**, **d**), Spinal cord at day 391 (**e**, **f**) after injection with gadodiamide, gadobutrol, and saline. *ns* indicates *p* > 0.05, *** indicates *p* < 0.001

To validate the genetic alteration, the expression of *Hmgb2* and *Sdk1* was determined by qRT-PCR and WB assays. After the 1-year washout, qRT-PCR analysis revealed higher gene expression levels of *Hmgb2* and *Sgk1* in brain tissues of the gadodiamide group compared to the saline group, with 4.9-fold higher *Hmgb2* and 4.4-fold higher *Sgk1*. No such significant differences were observed between the gadobutrol and saline groups. In spinal cord tissues, *Hmgb2* gene expression was 2.1-fold higher, and *Sgk1* expression was 7.1-fold higher in the gadodiamide group. While again, no significant differences were found between the gadobutrol and saline groups. At the end of injections on day 29, *Hmgb2* and *Sgk1* expression levels in brain tissues did not differ among the gadodiamide, gadobutrol, and saline groups (Fig. 5).

**Fig. 6:**
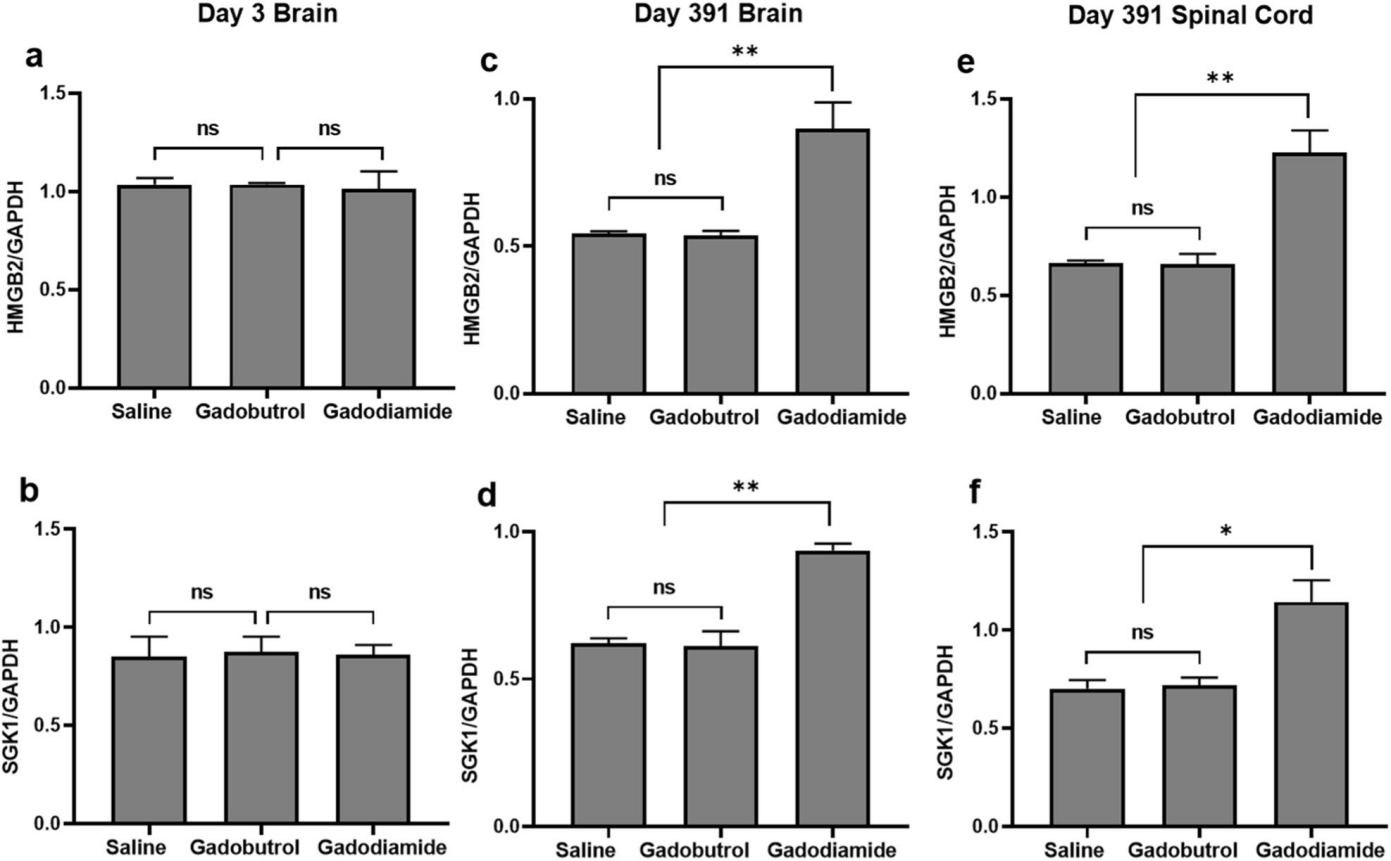
Relative protein levels of HMBG2 and SGK1. Brain at day 29 (**a**, **b**), brain at day 391 (**c**, **d**), Spinal cord at day 391 (**e**, **f**) after injection with gadodiamide, gadobutrol, and saline. *ns* indicates *p* > 0.05, * indicates *p* < 0.05, ** indicates *p* < 0.01

Similar trends were observed at the protein level by WB analysis (Supplemental Fig. S1). After the 1-year washout, HMGB2 protein levels were 52% higher in brain tissues and 57% higher in spinal cord tissues of the gadodiamide group compared to saline controls, while SGK1 protein was 2.5-fold higher both in the brain and in the spinal cord. In contrast, no significant differences in HMGB2 and SGK1 protein expression were observed in the brain and spinal cord between the gadobutrol and saline groups following the 1-year washout. Similarly, on day 29, there were no significant differences in HMGB2 and SGK1 protein levels among the gadodiamide, gadobutrol, and saline groups (Fig. 6).

In addition to WB and qRT-PCR experiments, immunohistochemical analysis was performed of mouse brain tissues after a 1-year washout, and the results are presented in Supplemental Figs. S2 and S3. These figures reveal a significant upregulation of HMGB2 and SGK1 expression in the brain tissues of mice treated with the linear GBCA gadodiamide when compared to both the saline control group and the group treated with the macrocyclic GBCA gadobutrol.

### Pathway enrichment analysis

Table 3 summarizes the results of the pathway enrichment analysis on the DEGs in the brain and spinal cord tissues between the gadodiamide and saline group after the 1-year washout. Compared to the saline group, the gadodiamide group showed significantly altered expression involved in the interferon-gamma signaling, indicating potential neuroinflammation responses induced by chronic gadodiamide retention.

**Table 3:** Enriched pathways of differentially expressed genes in brain and spinal cord

Tissues and enriched pathways	*p*-value
Brain	
NPAS4 regulates the expression of target genes	< 0.001
Transcriptional regulation by NPAS4	< 0.001
Interferon-gamma signaling	< 0.001
Cytokine signaling in the immune system	0.006
Interferon signaling	0.007
Generic transcription pathway	0.030
Spinal cord	
Insulin-like growth factor-2 mRNA binding proteins bind RNA	< 0.001
Gastrulation	0.002
Interferon-gamma signaling	0.003

## Discussion

Understanding the long-term effects of retained gadolinium in the CNS is paramount, given the widespread use of GBCAs in medical imaging and the potential safety implications for millions of patients. Our preclinical study indicates the retained gadolinium from linear GBCA (gadodiamide) significantly alters the interferon-gamma signaling pathway, a key mediator of inflammation, after a 1-year washout. We further validated representative DEGs, *Hmgb2* and *Sgk1*, using qRT-PCR and WB assays. To our knowledge, this is the first study to identify genetic and protein-level alterations in the CNS due to chronic gadolinium deposition.

Following repeated GBCA injections, T1-weighted hyperintensity was evident in the DCN of mice in gadodiamide group but not in gadobutrol group. Elevated gadolinium concentrations remained in the CNS tissues even after a 1-year washout. Previous studies have documented the slow clearance rate of gadodiamide from the brain, such as a 25% decrease over 1 year in rats [3], a 24% decrease over 1 year in mice [4], and a 15% decrease over 1 year in rats [5]. Our study corroborates these findings, showing a 21.2% decrease over 1 year in mice in the gadodiamide group, and almost all are washout in the gadobutrol group.

While chronic gadolinium retention in CNS tissues has not been linked with histopathological evidence of toxicity, transcriptomic analysis offers a sensitive tool to detect genetic alterations potentially preceding histological changes. Earlier studies found no significant differential gene expression in brain tissues after a brief washout period [9, 10], consistent with our observations. However, our data reveals that after a 1-year washout, chronically retained gadodiamide significantly modifies genes associated with interferon-gamma signaling pathways in both brain and spinal cord tissues. Gadolinium’s proinflammatory potential through induction of type I interferon signature *in vitro* has been documented [11, 12]. Among the dysregulated genes we identified, HGBM2 and SGK1 overexpression were confirmed using qRT-PCR and WB assays. HGBM2, a proinflammatory mediator, facilitates deoxyribonucleic acid−DNA processes and is upregulated in activated microglia post-neuronal injury [13, 14]. Similarly, SGK1 expression surges in the nervous system during neuroinflammation [15] and injury [16]. Given that neuroinflammation is the CNS innate immune response to harmful stimuli, our results underscore the potential long-term neurological implications of gadodiamide and other linear GBCAs. This is particularly concerning for multiple sclerosis patients, as linear agents have previously shown neurotoxicity in inflamed brains.

Earlier studies reported no neurochemical or behavioral effects linked to gadodiamide, except for pain hypersensitivity [2, 17] Our findings suggest that mechanical hyperalgesia was reversible after a 1-year washout, implying that transient pain hypersensitivity might not be attributed to chronically retained gadolinium. However, some studies noted a trend towards an anxiogenic effect in rats after gadodiamide injection [18, 19]. Elevated *SGK1* expression has been associated with increased anxiety- and depression-like behaviors in mice [15, 20], necessitating further research to ascertain if chronic gadolinium retention affects mood.

In the case of the gadobutrol group, the discrepancy between the transcriptomic detection of a few DEGs and the lack of significant expression changes in key genes, such as *Hgbm2* and *Sgk1*, likely reflects the minimal residual gadobutrol may not be enough to impact gene expression. Our study underscores the potential risks associated with the widespread use of GBCAs in clinical practice. The altered gene expression profiles observed in our model suggest that chronic exposure to gadolinium may have implications for neuroinflammatory processes and could contribute to neurological disorders. These findings emphasize the need for cautious use of GBCAs, particularly in patients with conditions that may predispose them to increased gadolinium retention. Furthermore, our results highlight the importance of developing strategies to mitigate the adverse effects of GBCAs and the potential for novel contrast agents with reduced doses and retention rates. Ultimately, this research serves as a foundation for future studies aimed at enhancing patient safety and improving clinical outcomes in imaging procedures.

Our study is not without limitations. Firstly, our research was conducted on healthy female mice, which cannot fully replicate the genomic condition of human patients. In fact, considering the limitations of resources and the number of experimental mice, choosing a single-sex mouse avoided the influence of gender differences on the gene expression level in neural tissues, enhancing the sensitivity and reliability of detecting gene alterations. Secondly, we did not confirm neuroinflammation using histochemical assays, though we did detect overexpression of *Hmgb2* and *Sgk1*, key proinflammatory mediators in the CNS. Thirdly, our study focused solely on gadodiamide and gadobutrol, limiting the generalizability across the GBCA class. Fourthly, we did not explore dose effects, and future research should investigate if single clinical dose injections influence CNS transcriptomes. Lastly, our techniques only identified transcriptomic changes in bulk tissue. Advanced methods like spatial transcriptomics and single-cell RNA sequencing are essential to pinpoint changes in specific CNS cell types and locations.

In conclusion, our research underscores that chronic gadolinium deposition after repeated injection of linear but not macrocyclic GBCA modifies gene expression in the CNS of mice, with DEGs, predominantly involved in neuroinflammation pathways. These insights necessitate further studies to elucidate the mechanisms and establish the long-term safety profile of linear GBCAs.

## Supplementary information

**Additional file 1**: **Figure S1**: WB bands of HMGB2 and SGK1 protein of gadodiamide, gadobutrol and saline group. (a): Brain at 29 days. (b) Brain at 391 days. (c) Spinal cord at 391 days. **Figure S2**: Immunohistochemistry of HMGB2 on mouse brain tissue at 391 days in saline, gadobutrol, and gadodiamide group. Scale bar = 50 μm. **Figure S3**: Immunohistochemistry of SGK1 on mouse brain tissue at 391 days in saline, gadobutrol and gadodiamide group. Scale bar = 50 μm. **Table S1**. DEGs shared by the brain and spinal cord of the gadodiamide groups after the 1-year washout (ordered alphabetically).

## Data Availability

The datasets generated during and/or analyzed during the current study are available from the corresponding author upon reasonable request.

## Abbreviations

CNS: Central nervous system
DCN: Deep cerebellar nuclei
DEGs: Differentially expressed genes
GBCA: Gadolinium-based contrast agent
HMGB2: High-mobility group box 2
ICP-MS: Inductively coupled plasma mass spectrometry
qRT-PCR: Quantitative reverse transcription polymerase chain reaction
SGK1: Serum/glucocorticoid-regulated kinase 1
SI: Signal intensity
WB: Western blot
