# Supplementary material for: Long-term effects of linear versus macrocyclic GBCAs on gene expression in the central nervous system of mice
Source: Eur Radiol Exp. 2025 Jan 10;9:3. doi: 10.1186/s41747-024-00546-x (PMC11723877; doi:10.1186/s41747-024-00546-x)
Supplement: Supplementary file 1 — Additional file 1: Figure S1: WB bands of HMGB2 and SGK1 protein of gadodiamide, gadobutrol and saline group. (a): Brain at 29 days. (b) Brain at 391 days. (c) Spinal cord at 391 days. Figure S2: Immunohistochemistry of HMGB2 on mouse brain tissue at 391 days in saline, gadobutrol, and gadodiamide group. Scale bar = 50 μm. Figure S3: Immunohistochemistry of SGK1 on mouse brain tissue at 391 days in saline, gadobutrol and gadodiamide group. Scale bar = 50 μm. Table S1. DEGs shared by the brain and spinal cord of the gadodiamide groups after the 1-year washout (ordered alphabetically). [file 41747_2024_546_MOESM1_ESM.pdf]

Long-term effects of linear versus macrocyclic GBCAs on gene expression in the central nervous system of mice

ELECTRONIC SUPPLEMENTARY MATERIAL

Eur Radiol Exp (2024) Wang C, Tang Y, Tang J, et al.

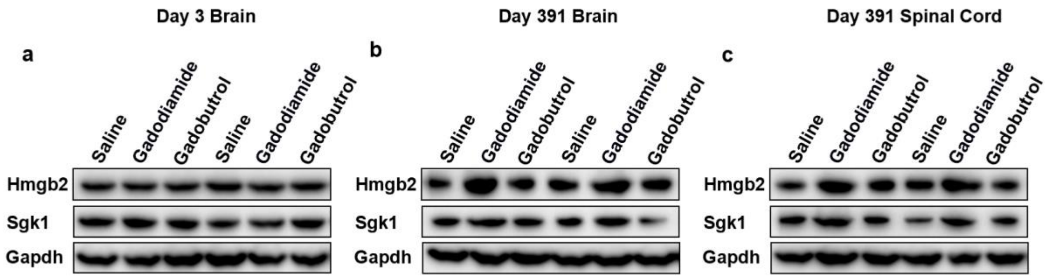

Figure S1: WB bands of HMGB2 and SGK1 protein of gadodiamide, gadobutrol and saline group. (a): Brain at 29 days. (b) Brain at 391 days. (c) Spinal cord at 391 days.

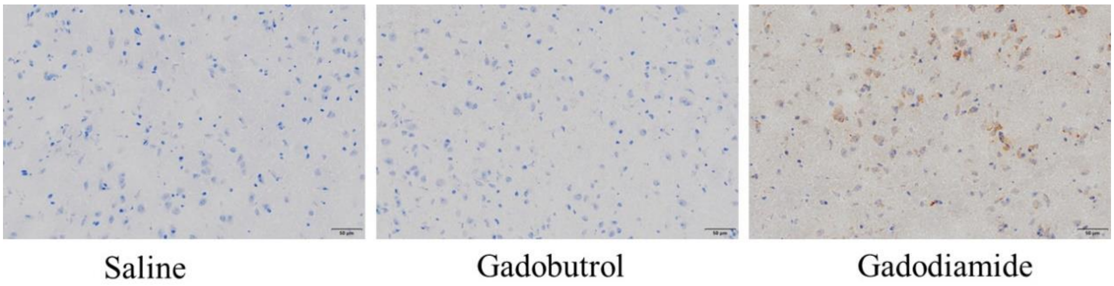

Figure S2: Immunohistochemistry of HMGB2 on mouse brain tissue at 391 days in saline, gadobutrol and gadodiamide group. Scale bar = 50 μm.

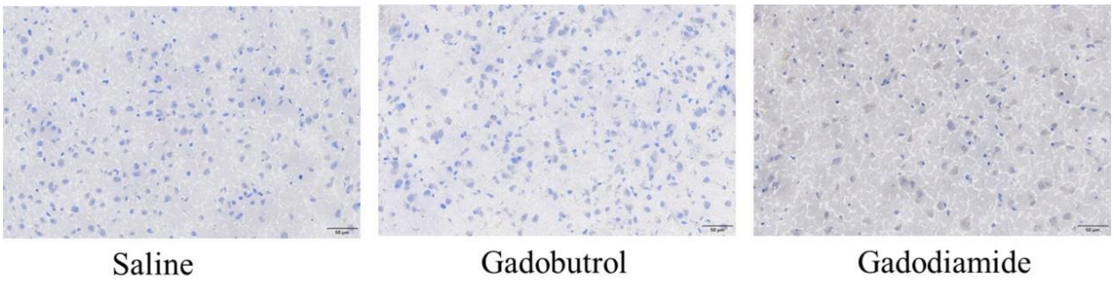

Figure S3: Immunohistochemistry of SGK1 on mouse brain tissue at 391 days in saline, gadobutrol and gadodiamide group. Scale bar = 50 μm.

Table S1. DEGs shared by the brain and spinal cord of the gadodiamide groups after the 1-year washout (ordered alphabetically)

Gene	Full Name
Arrdc2	Arrestin Domain Containing 2
Bdkrb2	Bradykinin Beceptor B2
Clk1	CDC-Like Kinase 1
Fam110d	Family With Sequence Similarity 110 Member D
Foxf1	Forkhead Box F1
Gbp4	Guanylate Binding Protein 4
Gm527	Predicted Gene 527
Gp1ba	Glycoprotein 1b Platelet Subunit Alpha
Hbb-bt	Hemoglobin, Beta Adult T Chain
Hmgb2	High Mobility Group Box 2
Map3k6	Mitogen-Activated Protein Kinase Kinase Kinase 6
Rrh	Retinal Pigment Epithelium-Derived Rhodopsin Homolog
Sgk1	Serum/Glucocorticoid Regulated Kinase 1
Sox18	SRY-Box Transcription Factor 18
Tm6sf2	Transmembrane 6 Superfamily Member 2
Wfikkn1	WAP, Follistatin/Kazal, Immunoglobulin, Kunitz And Netrin Domain Containing 1
Zfp658	Zinc Finger Protein 658
